# Purification and Characterization of a Unique Pectin Lyase from *Aspergillus giganteus* Able to Release Unsaturated Monogalacturonate during Pectin Degradation

**DOI:** 10.1155/2014/353915

**Published:** 2014-12-31

**Authors:** Danielle Biscaro Pedrolli, Eleonora Cano Carmona

**Affiliations:** ^1^Department of Bioprocess and Biotechnology, School of Pharmaceutical Sciences, Universidade Estadual Paulista (UNESP), Rodovia Araraquara-Jaú km 1, 14801-902 Araraquara, SP, Brazil; ^2^Department of Biochemistry and Microbiology, Biosciences Institute, Universidade Estadual Paulista (UNESP), Avenida 24A 1515, 13506-900 Rio Claro, SP, Brazil

## Abstract

A pectin lyase, named PLIII, was purified to homogeneity from the culture filtrate of *Aspergillus giganteus* grown in submerged culture containing orange peel waste as carbon source. PLIII was able to digest apple pectin and citrus pectins with different degrees of methyl esterification. Interestingly, the PLIII activity was stimulated in the presence of some divalent cations including Pb^2+^ and was not significantly affected by Hg^2+^. Like other pectin lyases, PLIII is stimulated by but is not dependent on Ca^2+^. The main soluble product released during the degradation of pectic substances promoted by the PLIII is compatible with an unsaturated monogalacturonate. PLIII is a unique enzyme able to release unsaturated monogalacturonate as the only soluble product during the degradation of pectic substances; therefore, PLIII was classified as an exo-pectin lyase. To our knowledge, this is the first characterization of an exo-pectin lyase. The PLIII described in this work is potentially useful for ethanol production from pectin-rich biomass, besides other common applications for alkaline pectinases like preparation of textile fibers, coffee and tea fermentation, vegetable oil extraction, and the treatment of pulp in papermaking.

## 1. Introduction

Pectinases production occupies about 10% of the overall manufacturing of enzyme preparations. These enzymes are traditionally used by food industry in the production of juices, fruit drinks, and wines [1] and also can be used in the pretreatment of waste water from vegetable food-processing that contains pectin residues; in the processing of textile fibers, such as flax, jute, and hemp; in coffee and tea fermentation; in vegetable oil extraction; and in the treatment of paper pulp [1–4]. Recently, pectinases have been applied in studies on ethanol production from pectin-rich biomass [5, 6]. Residues such as apple pomace, citrus peel waste, and sugar beet pulp are industrial waste left after fruits or vegetables have been processed for juice or sugar production. These pectin-rich biomasses are an abundant and widely underused resource [7].

Pectin lyase (PL; EC 4.2.2.10) is a pectinolytic enzyme that catalyzes the cleavage of pectin, preferentially highly esterified pectin, producing unsaturated methyloligogalacturonates through transelimination of glycosidic linkages. PLs are classified into the polysaccharide lyase family 1 (CAZy database). Fungal PLs usually present optimum activity in acid and neutral medium, while those from bacteria are more active in alkaline medium. PLs do not present an absolute requirement for Ca^2+^, but they are stimulated by this and other cations [3]. Up until now, all described pectin lyases are endo-PLs [8]. Van Alebeek et al. [9] conducted a detailed study about the mode of action of the pectin lyase A from* Aspergillus niger* which produces mono-, di-, tri-, and tetragalacturonates, besides unsaturated di-, tri-, and tetragalacturonates from methyloligogalacturonates; unsaturated monogalacturonates were not identified in the reaction products in any assay.

The analysis of enzyme activity in crude extract does not indicate either an isolated action or the presence of a multienzymic system working in synergy on the substrate degradation. The characterization of purified enzymes is an important research line since it provides discrimination between the enzymic complex components with respect to substrate degradation mechanism, optimum activity conditions, and enzyme synthesis regulation.

*Aspergillus giganteus* produces a pectinolytic complex which includes at least one polygalacturonase [10] and three pectin lyases. In order to access the characteristics of the pectin lyase enzymatic complex in* A. giganteus*, the enzyme system was produced in submerged fermentation containing solid waste from juiced oranges and separated into three active fractions called PLI, PLII, and PLIII. The major component of the complex (PLIII) was purified until electrophoretic homogeneity and had its main properties determined. Orange peel waste is an agroindustrial residue abundantly produced in Brazil by the orange juice industry and its utilization for PL production leads to an increase in the enzyme yield together with a reduction in the process cost; moreover, it adds value to the industrial waste.

## 2. Material and Methods

### 2.1. Materials

Polygalacturonic acid sodium salt, D-galacturonic acid monohydrate, digalacturonic acid, trigalacturonic acid, citrus pectin (esterification degree of 34% and 90%), apple pectin (esterification degree of 75%), and chromatographic resins were all purchased from Sigma. Citrus pectin (esterification of 72%) was kindly donated by CP Kelco (Limeira, SP, Brazil).

### 2.2. Culture and Maintenance of the Fungal Strain

*Aspergillus giganteus* strain CCT 3232 is maintained in the culture collection of the “Fundação Tropical de Pesquisa e Tecnologia André Tosello” (Brazil). The strain was previously isolated from soil of the Brazilian Atlantic Forest, at Peruíbe, São Paulo State. Spores were collected after 7 days of growth on Vogel agar medium slopes. Vogel liquid medium enriched with orange peel waste was used to grow* A. giganteus* and to induce pectinases production. The orange peels were washed with distilled water, dried at 110°C, and finally ground up using a grain mill. Inoculum preparation and enzyme production were carried out according to the conditions established before [11].

### 2.3. Enzyme Assay

The pectin lyase activity was assayed as described by Pitt [12]. The enzyme solution was incubated in 50 mM glycine-NaOH buffer, pH 8.5, 1% citrus pectin (72% methyl-esterified), and 0.01 M CaCl_2_ at 50°C. The reaction was stopped by addition of 0.6 mL ZnSO_4_ × 7 H_2_O (9%, w/v) followed by 0.6 mL NaOH (0.5 M). The reaction mixture was centrifuged at 3,000 ×g for 10 min and 5.0 mL of the supernatant was transferred to a fresh tube. Next, 3.0 mL of 0.04 M thiobarbituric acid, 1.5 mL of 0.1 M HCl, and 0.5 mL of water were added to the assay mixture. The final solution was incubated in a boiling water bath for 30 min and cooled. The accumulation of the reaction products was followed photometrically at *λ* = 550 nm. One unit of activity was defined as the amount of enzyme causing a 0.010 rise in absorbance units per hour. The specific activity was calculated with respect to the protein content.

### 2.4. Protein Determination

Protein content was quantified by the Lowry method using bovine serum albumin as standard or by direct absorbance measurement at 280 nm.

### 2.5. Enzyme Purification

Three chromatography steps were used to isolate the main pectin lyase. Before each chromatography step the samples were dialyzed against the same buffer used to equilibrate the columns: (1) DEAE-Sephadex A-50 column equilibrated with 50 mM imidazole-NaOH, pH 6.0, eluted with a linear gradient from 0.0 to 1.0 M NaCl; (2) CM-Sephadex C-50 column equilibrated with 50 mM sodium acetate buffer, pH 5.5, eluted with a linear gradient of NaCl up to 0.5 M; and (3) Sephadex G-100 column equilibrated with 50 mM ammonium acetate, pH 6.8.

### 2.6. Influence of pH and Temperature on Enzyme Activity

The purified pectin lyase was incubated with the substrate at pH values ranging from 6.5 to 10 at 50°C. Three different buffer systems were used, namely, 50 mM imidazole-NaOH, pH 6.5; 50 mM Tris-HCl from pH 7.0 to 8.0; and 50 mM glycine-NaOH from pH 8.5 to 10.0. To determine the optimal temperature for PL activity, the enzyme was incubated with the substrate at temperatures ranging from 30°C to 60°C.

### 2.7. Enzyme Stability

The thermal stability of the PL activity was assayed as residual activity after incubating the pure enzyme in 50 mM glycine-NaOH, pH 8.5, at 40, 45, and 50°C in the absence of substrate. The pH stability was determined as the residual PL activity after the purified enzyme had been incubated for 24 h at 4°C without substrate in the following 50 mM buffer systems: glycine-HCl (pH 2.5–3.5), sodium acetate (pH 4.0–5.5), imidazole-NaOH (pH 6.0–6.5), Tris-HCl (pH 7.0–9.0), and glycine-NaOH (pH 9.5–10.0).

### 2.8. Gel Electrophoresis

The homogeneity and molecular mass of the purified PL were checked by electrophoresis on 8–18% polyacrylamide gels (sodium dodecyl sulfate-polyacrylamide gel electrophoresis, SDS-PAGE) according to Laemmli [13]. Protein bands in the gels were stained with Coomassie brilliant blue G-250.

### 2.9. Thin Layer Chromatography

Thin layer chromatography (TLC) was used to identify the soluble products of pectin degradation. Activity assay samples were inactivated with 40 mM NaOH and 0.7% (w/v) ZnCl_2_ and centrifuged at 10,000 ×g. Supernatants were spotted on silica gel 60 aluminum sheets (15 × 10 cm; Merck, Germany). Chromatography was performed twice using the ascending method with ethyl acetate : acetic acid : formic acid : water (9 : 3 : 1 : 4) as solvent system. For visualization of spots dried plates were sprayed with 0.2% (w/v) orcinol dissolved in 10% sulfuric acid : methanol (1 : 9 v/v) followed by heating at 105°C for 5 min [14].

### 2.10. Substrate Specificity

The purified PL was incubated with different substrates (citrus pectin with three different methylation degrees of the carboxyl groups, 34%, 72%, and 90%; apple pectin; and polygalacturonic acid) at fixed concentration (1%) in 50 mM glycine-NaOH, pH 8.5.

### 2.11. Kinetic Parameters

The kinetic constants *K*_*m*_ and *V*_max⁡_ of the purified enzyme were calculated by fitting the reaction rate data at different substrate concentrations to a linear regression on Lineweaver-Burk double-reciprocal plots. The kinetic parameters were determined twice using citrus pectin with 34% and 72% degree of esterification as substrates under optimal assay conditions.

### 2.12. Influence of Metal Ions and Other Compounds on Enzyme Activity

The effect of a number of cations, denaturants, and chelating agents on enzyme activity was tested in the reaction assay. PL was assayed in the presence of each substance at 2 mM. Before the assay, the enzyme solution was dialyzed against 50 mM glycine-NaOH, pH 8.5.

## 3. Results and Discussion

**Figure 1:**
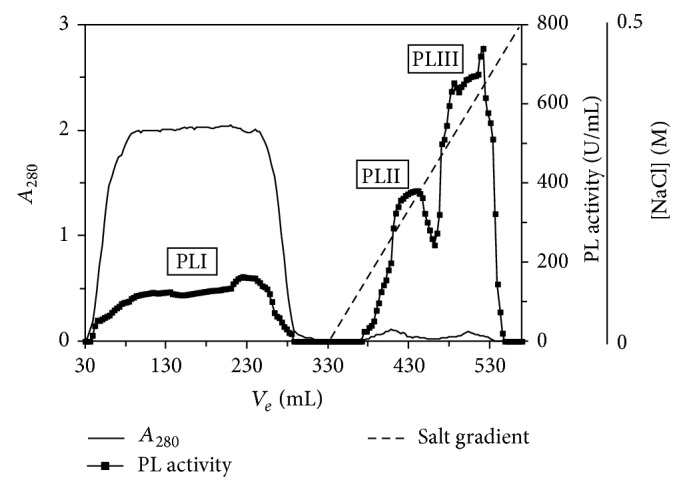
Cation-exchange chromatography on CM-Sephadex A-50 column of the pectin lyase complex produced by* Aspergillus giganteus*. Solid line: absorbance at 280 nm; filled squares: PL activity; broken line: salt gradient.

*A. giganteus* secreted at least three isoforms of pectin lyase in the cultured conditions. The PL showing higher total activity was purified after three steps. In the first step, DEAE-Sephadex A-50 chromatography, the proteins responsible for PL activity were eluted together with a linear gradient from 0.0 to 1.0 M NaCl. In the second step, CM-Sephadex C-50 chromatography, proteins were eluted with a linear gradient up to 0.5 M NaCl, and three active peaks were detected, called PLI, which did not bind to the column, PLII, and PLIII (Figure 1). The last chromatographic step, gel-filtration, and the enzymatic characterization were performed only with PLIII, which seemed to be the most abundant isoform secreted by* A. giganteus* in the cultured conditions.

**Figure 2:**
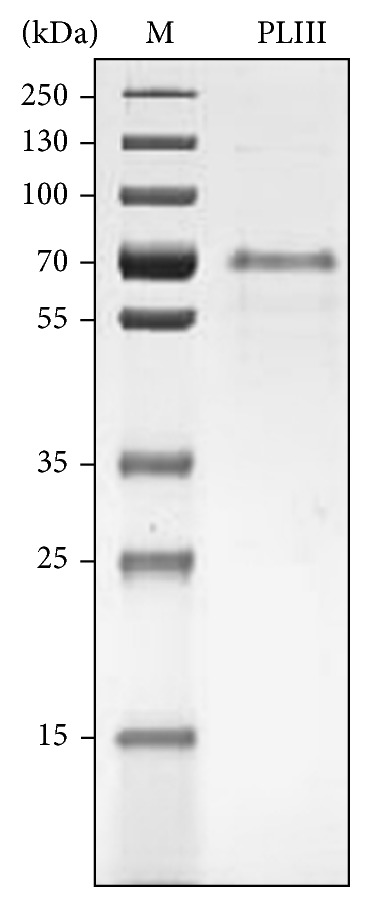
Pure PLIII preparation analyzed by SDS-PAGE. Lanes: M: molecular mass standard proteins and PLIII: major pectin lyase after the Sephadex G-100 column step.

After three chromatographic steps, the PLIII presented electrophoretic homogeneity (Figure 2) and purification fold of 47.8 with recovery of 12.4% (Table 1). The purified PLIII had its molecular weight estimated in 71 kDa by SDS-PAGE (Figure 2) and 63 kDa by gel-filtration chromatography.

**Figure 3:**
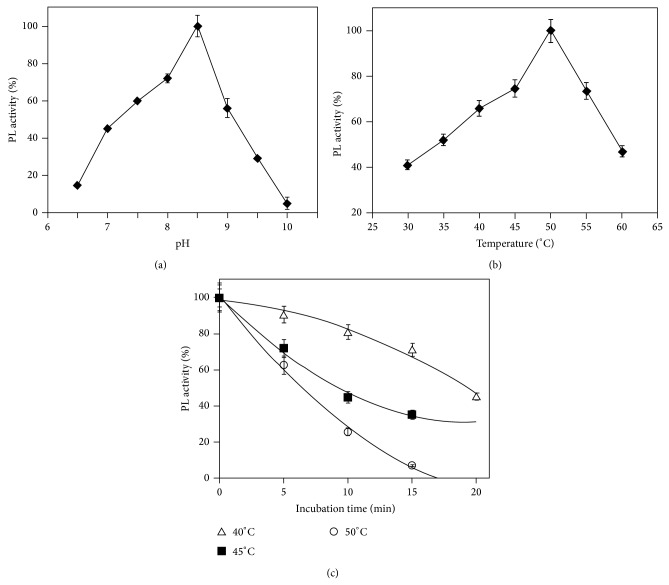
Influence of pH (a) and temperature (b) on PLIII activity and the thermal inactivation (c) of the purified PLIII from* A. giganteus*. The vertical bars indicate standard deviation (SD) of the mean calculated for three replicates.

The pH dependence of the PLIII activity is shown in Figure 3(a). The enzyme exhibited lyase activity in neutral and alkaline pectin solutions with optimal activity at pH 8.5. Maximal activity in alkaline medium seems to be an atypical behavior for pectin lyases, since most of the microbial pectin lyases described in the literature have maximal activity at pH values ranging from 5.0 to 7.0. Alkaline pectinases are predominantly pectate lyases, which preferentially digest nonmethylated pectin and are dependent on calcium ions [15].

Despite its preference for alkaline pH values for action, PLIII showed the highest stability when incubated in acid to neutral solutions (pH ranging from 3.0 to 7.0). Under these conditions the enzyme solutions kept more than 90% lyase activity after 24 h incubation at 4°C.

When incubated in diverse temperatures in the presence of citrus pectin as substrate, the PLIII showed maximal activity at 50°C (Figure 3(b)), in agreement with other microbial pectinases already characterized [3, 11]. The thermal stability of the enzyme was assayed immediately after its incubation without substrate at three different temperatures (Figure 3(c)). In the absence of substrate the PLIII was reasonably stable at 40°C, keeping 70% of its activity after 15 min, but at 50°C the enzyme lost its activity fast, with a half-life of 9 min. Nevertheless, the* A. giganteus* PLIII was more stable than the commercially available enzymes Rapidase C80 and Pectinase CCM [16].

**Figure 4:**
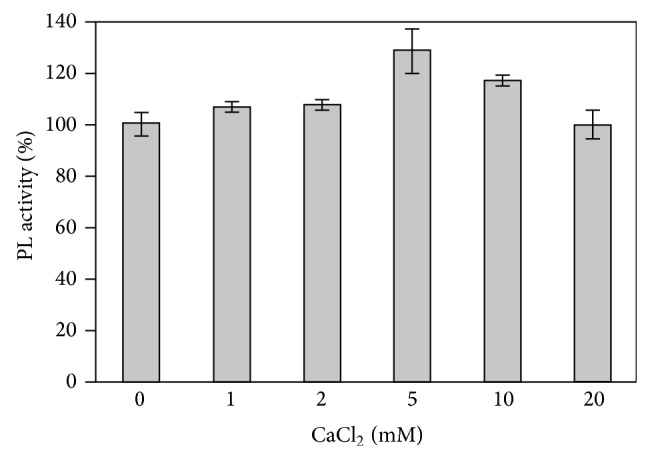
Effect of Ca^2+^ ions on the purified PLIII activity. The vertical bars indicate standard deviation (SD) of the mean calculated for three replicates.

Pectic substances tend to form a gel structure under certain conditions. Various factors determine gelling properties including temperature, pectin type, esterification degree, acetylation degree, pH, and presence of sugar and other solutes. However, the main gelling inducer seems to be calcium ions, which interact with pectin unesterified carboxyl groups cross-linking the homogalacturan portions of the pectin molecule and lead to the formation of a three-dimensional crystalline network in which water and solutes are trapped [17]. To assess the influence of the pectin gelling degree and also the direct effect of Ca^2+^ on the PLIII activity, the enzyme activity was assayed in the presence of different CaCl_2_ concentrations (Figure 4). A stimulatory effect was observed when Ca^2+^ was present in concentrations up to 10 mM, reaching the highest effect at 5 mM. Even without any Ca^2+^ addition the enzyme showed high activity level. Calcium independent action is characteristic of the pectin lyase enzyme group, which is stimulated by but is not dependent on calcium ions [3, 11].

Pectic substances are complex high molecular mass glycosidic polymers formed by D-galacturonic acid which can be acetylated and/or methyl-esterified. The complex structure may also contain other sugars like rhamnose, galactose, arabinose, and xylose. Different degrees of methyl esterification are found in pectins depending on the plant source and also variation in the methyl esterification level can be found along the same polymer [17]. Microbial pectinases have evolved to specifically target each kind of pectic polysaccharide [3, 11]. To determine the specificity of the PLIII activity, the enzyme was assayed with citrus pectins exhibiting different levels of methyl esterification from 0 to 90% and with apple pectin (75% methyl esterification degree) (Table 2). The PLIII was active on substrates with degrees of methyl esterification between 34% and 90% and was better at digesting the 34% and 72% esterified pectins from citrus. Although having a similar esterification level as compared to the best digested citrus pectin substrate, the apple pectin did not show the same digestibility. Possible differences in the pattern of esterification along the pectin polymer could explain this result. Methyl groups seem to be important to the PLIII binding/digestion process since nonesterified pectin (sodium polygalacturonate) was not accepted as substrate.

Very similar degradation rates were observed for citrus pectins with 34% and 72% methyl esterification degrees. Therefore, the kinetic parameters *V*_max⁡_ and *K*_*m*_ were determined for PLIII in the presence of both substrates to assess possible minor differences in the degradation process of these pectins. The kinetic parameters indicate that the PLIII slightly favors the degradation of the 34% over the 72% methyl-esterified pectin (Table 3).

**Figure 5:**
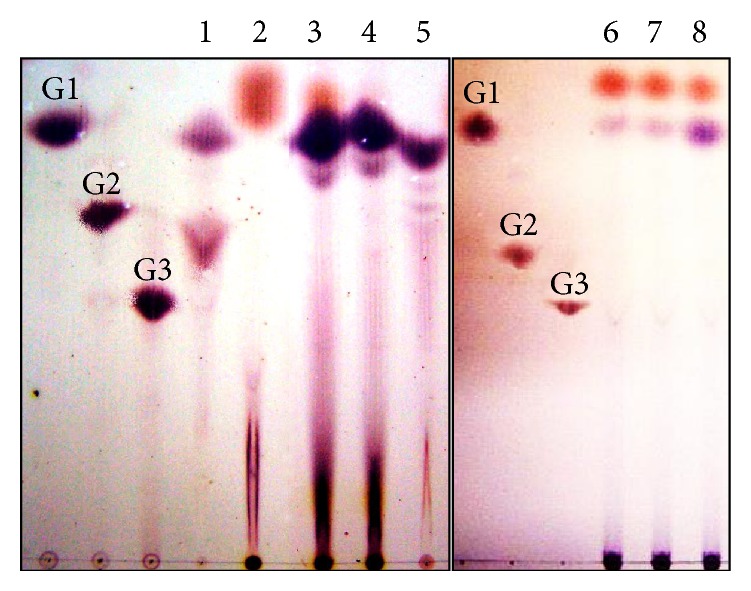
Degradation pattern of pectic substances by the purified PLIII from* A. giganteus*. Each lane corresponds to one substrate submitted to enzymatic degradation. Lane 1: digalacturonic acid, lane 2: polygalacturonic acid, lane 3: citrus pectin MD 72%, lane 4: citrus pectin MD 34%, lane 5; apple pectin MD 75%, and lanes 6, 7, and 8; citrus pectin MD 72% after 30 min, 2 h, and 24 h incubation, respectively. Standards: G1: monogalacturonic acid, G2: digalacturonic acid, and G3: trigalacturonic acid. The upper spots in lanes 2, 3, 6, 7, and 8 result from the alkaline hydrolysis of the substrate due to the reaction conditions also seen in the controls without enzyme addition (data not shown).

Pectinases generally digest pectins by catalyzing random cleavage of internal polymer linkages (endo-pectinases) or by catalyzing hydrolytic cleavage at substrate nonreducing end producing monogalacturonate or digalacturonate (exo-pectinases). The degradation pattern of five different types of pectic substances was analyzed in thin layer chromatography (TLC) after 10 h digestion with PLIII and compared with the standards: galacturonic acid (G1), digalacturonic acid (G2), and trigalacturonic acid (G3) (Figure 5). As quoted before, the enzyme was not able to degrade sodium polygalacturonate (Figure 5, lane 2). The spot observed for this sample, as well as the upper spots in lanes 3, 6, 7, and 8, results from the alkaline hydrolysis of the substrate due to the reaction conditions also seen in the control assays without enzyme (data not shown). The main soluble product released by the PLIII during degradation of citrus and apple pectins was compatible with G1 standard, probably unsaturated monogalacturonate (Figure 5, lanes 3, 4, and 5). The enzyme was also able to degrade digalacturonate, releasing monogalacturonate (Figure 5, lane 1). To determine whether the released product could be a saturated galacturonate generated by hydrolysis, the reducing sugar content in the reaction samples was measured by Miller's method. No reducing sugar increase was found in any reaction, validating the PLIII classification as an exo-pectin lyase capable of releasing unsaturated monogalacturonate from pectin molecules.

To determine whether the pectin degradation pattern observed in the previous assay was time dependent, 72% methyl-esterified citrus pectin was incubated in the presence of PLIII for 30 min, 2 h, and 24 h and the soluble degradation products were thereafter analyzed in TLC. The only difference observed on pectin degradation after increasing incubation time was higher product accumulation, without any change in the degradation pattern (Figure 5, lanes 6, 7, and 8).

Since pectic substrates are susceptible to undergoing changes in their physical and chemical properties in the presence of salts and other substances, the effect of a number of cations, a denaturant, and chelating agents on enzyme activity was tested in the reaction assay. PLIII was assayed in the presence of each substance at 2 mM (Table 4). Although calcium ions are widely described as pectinases activators, the PLIII accepted some other cations besides Ca^2+^. The divalent cations Co^2+^, Pb^2+^, Ba^2+^, Mg^2^, and Zn^2+^ worked even better as activity enhancers at 2 mM than Ca^2+^. Cobalt ion was found to strongly stimulate PLIII activity to 153% as compared to the reaction without additives. Notably, PLIII is resistant to the toxic ions Hg^2+^ and Pb^2+^; furthermore, the last one was found to enhance the enzyme activity to 134%. The positive effect that some divalent cations exhibit over pectin lyases activity is supposed to be a result of their interaction with the pectic substrates causing gelatinization [11, 17, 18] and, possibly, the potential toxic effects of lead and mercury ions on the enzyme were prevented due to their capture in the three-dimensional crystalline network of the pectin. Interestingly, Pb^2+^, Co^2+^, and Zn^2+^ are commonly described as inhibitors of pectin lyase activity and Hg^2+^ is mostly known to completely inactivate PLs [19–22].

The mild negative effect on PLIII activity caused by chelating agents, citrate and EDTA, evidenced the inducing effect of cations on the enzyme activity. Even though no cation was added to the reaction, trace amounts of contaminant Ca^2+^ ions might be present in the pectin substrate.

Among the tested substances only SDS (sodium dodecyl sulfate) severely reduced the PLIII activity, possibly by disrupting important hydrophobic interactions within the enzyme structure.

## 4. Conclusion

The global call for using renewable raw materials turned the attention to biopolymers like cellulose, hemicellulose, lignin, and pectin and consequently to the corresponding degrading enzymes, cellulases, xylanases, lignin peroxidases, and pectinases. With poor nutritional properties and treated as waste material in the past, plant fibers present in fruit peel and sugar cane bagasse have been extensively studied for their potential as raw material mainly for the biofuel industry [5–7]. The discovery of new cell wall degrading enzymes with diverse activity specificities and product yields can allow access to the sugars present in the biopolymers structure making their use in industrial processes like ethanol production practicable.

We report here a new pectinolytic enzyme specialized in the degradation of methyl-esterified pectins under neutral and alkaline conditions. The enzyme is not affected by possible ions contamination, including the toxic Pb^2+^ and Hg^2+^; rather, it is stimulated by some common cations. Additionally, we describe the first pectinase able to digest methyl-esterified pectins to monomers without the need for other enzymes. Notably, galacturonate and Δ4,5-unsaturated galacturonate can be metabolized by the engineered* E. coli* KO11 for ethanol production [5, 23]. Furthermore, the pectin lyase described in this work is potentially useful for the treatment of waste water from vegetable-processing, textile fiber preparation, coffee and tea fermentation, vegetable oil extraction, and the treatment of pulp in papermaking.

The preference for esterified pectins and the nondependence on Ca^2+^, despite its enhancing effect, indicates that the purified PLIII can be classified as a pectin lyase. Additionally, the enzyme releases only one soluble product compatible with an unsaturated monogalacturonate, showing an exo-mode of action. These findings represent the first description of an exo-pectin lyase. Our next step will be to identify, clone, and express in* E. coli* the genes of* A. giganteus* that code for all three pectin lyases identified in this work.

## Figures and Tables

**Table 1:** Pectin lyase purification steps.

Purification step	Total activity (U)	Protein (mg)	Specific activity (U/mg of protein)	Yield (%)	Purification fold
Crude extract	181,300.0	230.9	785.2	100.0	1.0
DEAE-Sephadex A-50	157,529.0	54.3	2,901.1	87.0	3.7
CM-Sephadex C-50					
PLI	25,034.4	34.6	724.4	13.8	0.9
PLII	18,012.0	2.5	7,125.0	9.9	9.1
PLIII	52,540.0	1.4	37,528.5	29.0	47.8
Sephadex G-100 (PLIII)	22,500.0	0.6	37,500.0	12.4	47.8

**Table 2:** Substrate specificity of PLIII from *A. giganteus*.

Substrate	PG (%)
Citrus pectin (MD^*^ 72%)	100.0 ± 3.0
Citrus pectin (MD 34%)	99.1 ± 3.0
Apple pectin (MD 75%)	59.5 ± 4.7
Citrus pectin (MD 90%)	22.0 ± 5.5
Sodium polygalacturonate	0.0

^*^MD: methylation degree of the carboxyl groups of pectin.

**Table 3:** Kinetic parameters of PLIII from *A. giganteus*.

Kinetic parameter	Citrus pectin (MD^*^ 72%)	Citrus pectin (MD 34%)
*V*_max⁡_ (U·mg^−1^·min^−1^)	1,111.1	1,428.6
*K*_*m*_ (mg·mL^−1^)	4.9	4.6

^*^MD: methylation degree of the carboxyl groups of pectin.

**Table 4:** Influence of substances on PLIII activity from *A. giganteus*.

Substance (2 mM)	PL activity (%)^*^
CoCl_2_	153.6 ± 6.6
Pb(CH_3_COO)_2_	134.7 ± 3.2
BaCl_2_	132.4 ± 10.4
MgSO_4_	125.8 ± 12.6
ZnSO_4_	116.9 ± 4.8
NaCl	110.6 ± 5.6
CaCl_2_	107.4 ± 2.8
HgCl_2_	94.0 ± 8.9
MnSO_4_	88.5 ± 3.7
Sodium citrate	96.6 ± 0.6
EDTA	85.8 ± 7.4
DTT	102.4 ± 3.0
*β*-Mercaptoethanol	78.8 ± 5.0
Iodoacetic acid	75.1 ± 2.3
PMSF	67.2 ± 2.5
SDS	6.4 ± 0.9

^*^Control: 100% ± 6.1.
